# Enhanced perfusion of elliptical wound closures using a novel adhesive suture retention device

**DOI:** 10.1002/hsr2.364

**Published:** 2021-09-14

**Authors:** Allison Stoecker, William Lear, Karsten Johnson, Jared Bahm, Jamie J. Kruzic

**Affiliations:** ^1^ Departments of Dermatology and Dermatologic Surgery Silver Falls Dermatology Corvallis Oregon USA; ^2^ Jared Bahm Consulting Albany Oregon USA; ^3^ School of Mechanical and Manufacturing Engineering University of New South Wales (UNSW Sydney) Sydney New South Wales Australia

**Keywords:** adhesive retention suture device, perfusion, wound closure

## Abstract

**Background and Aims:**

The purpose of this investigation was to test the hypothesis that a novel adhesive retention suture device (ARSD) can increase perfusion at elliptical wound closures by distributing stress away from the suture site.

**Methods:**

Stress in the skin around a suture both with and without support from an ARSD was evaluated using a finite element model. A single‐center, randomized split‐scar comparison trial using laser speckle contrast analysis was used to quantify the perfusion at elliptical wound closures in human patients both with and without an ARSD.

**Results:**

The finite element model revealed that the ARSD promoted load transfer to the skin over a larger area, thus reducing the local stress and deformation in the skin around the suture site. Results from the split‐scar study showed a mean improvement of 25% perfusion units with the ARSD, and the improvement was statistically significant (*p* = 0.002).

**Conclusion:**

The reduction in local stress and enhanced perfusion around the suture site reveals the potential benefit of using an ARSD to enable more efficient healing by avoiding complications associated with both low perfusion and skin tearing, such as dehiscence, infection, and cheese wiring.

**Abbreviations and Acronyms**
ARSD: adhesive retention suture device
FEA: finite element analysis
LASCA: laser speckle contrast analysis
PETG: glycol‐modified polyethylene terephthalate
PUs: perfusion units
ROI: region of interest
TGFβ: transforming growth factor β
TOI: time of interest

## 1 INTRODUCTION

Normal wound healing is usually described in four successive phases: hemostasis, inflammation, proliferation, and remodeling.^1^ The *hemostatic* phase involves both the clotting cascade and influx of platelets during the initial 2 to 3 days. In the *inflammatory* phase, neutrophils and macrophages release key growth factors that activate fibroblasts whose main role during the *proliferative* phase is to lay down a new extracellular matrix, which is the key source of strength in the skin. Most of the wound strength is acquired during this phase, which typically lasts 1 to 6 weeks after injury. Neovascularization is also necessary during the proliferative phase for expanding the existing capillary network to nourish the other activities. In the final *remodeling* phase, wound contraction and strength acquisition continue, and the final wound strength typically achieves approximately 70% to 80% of uninjured skin.^2^, ^3^

Adequate perfusion is required to supply the cellular and metabolic demands required for the wound healing phases.^4^ Poor perfusion is associated with complications including wound dehiscence, infections, and delayed healing.^5^, ^6^, ^7^ Measurements of perfusion have proven to be important prognostic factors in the clinical management of wound healing, with one example being the ankle‐brachial index for the treatment of chronic lower extremity wounds.^8^ Laser speckle contrast analysis (LASCA) is another effective method to measure perfusion by real‐time camera imaging of a defined area over time. LASCA has been successfully used to monitor healing after burns and radiation therapy and is able to predict the need for excision and grafting.^9^, ^10^ Furthermore, LASCA measurements of perfusion have proven to correlate well with laser Doppler measurements while enabling faster measurements over larger areas.^9^, ^10^

Recently, an adhesive retention suture device (ARSD) has been reported to aid in the closure of fragile and high‐tension wounds.^11^ The ARSD has been shown to bolster skin strength and allow for closure of wounds even in situations where normal sutures had already torn the skin.^11^ Additionally, clinical observations have shown less skin blanching of the skin with the use of the ARSD in high‐tension wound closure. Here we hypothesize that the efficacy of the device is related to how stress is redistributed at the wound edge to create enhanced perfusion relative to a suture without the device. To test this hypothesis, we have developed a finite element analysis (FEA) model to assess the stress redistribution caused by the ARSD when it is added to a suture. We then examined the effect of the ARSD on perfusion in human patients when used with a nylon‐interrupted suturing pattern.

## 2 MATERIALS AND METHODS

### 2.1 Finite element model

**FIGURE 1:**
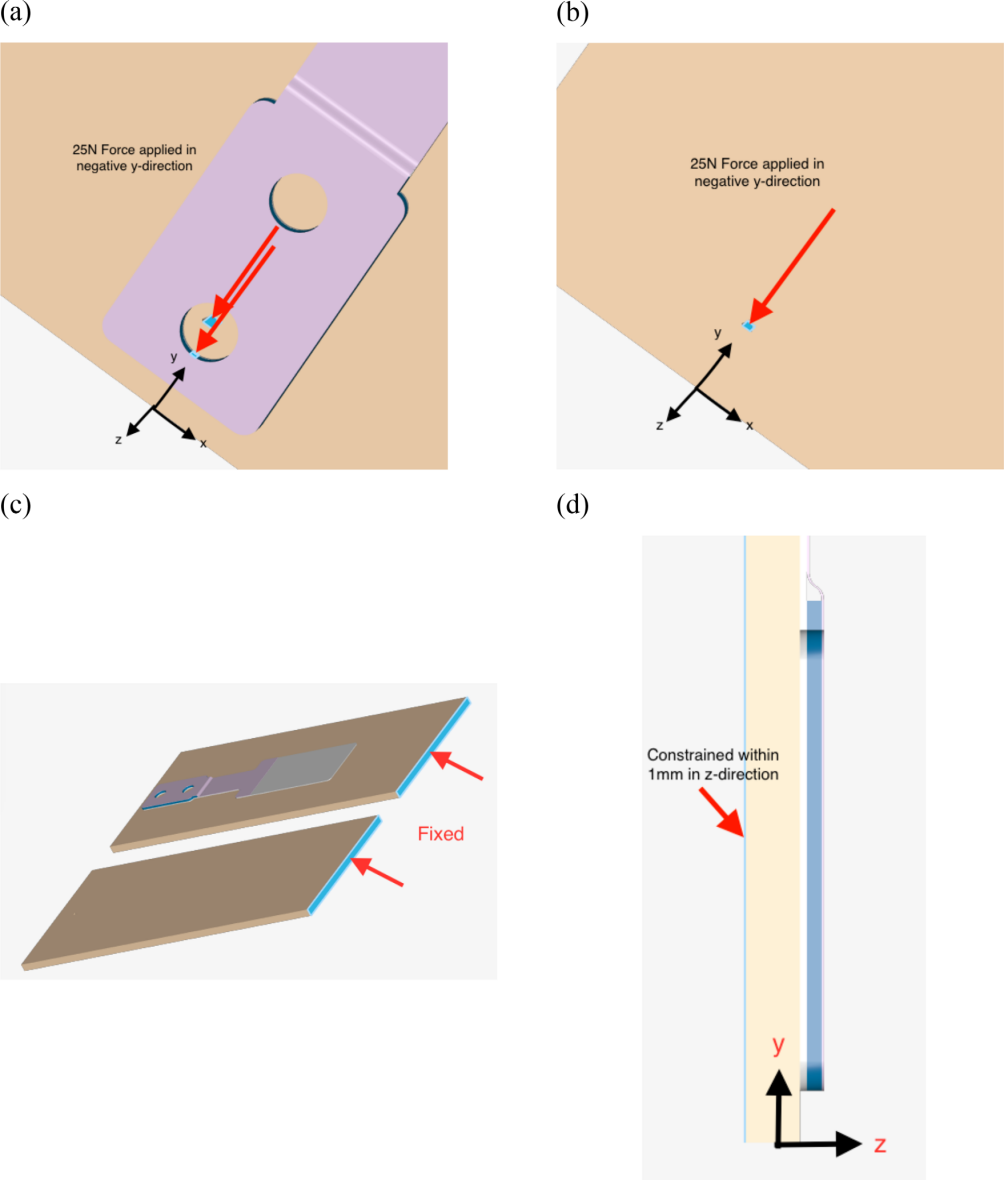
Solid model used for the finite element analysis with the x‐axis defined as parallel to the wound, the y‐axis defined along the suture loading direction, and the z‐axis defined as perpendicular to the skin surface. (a) and (b) show the configuration of the simulated skin samples with and without the ARSD, respectively. (c) shows the fixed displacement boundary condition and (d) depicts a cross‐sectional view of the y‐z plane where displacement was constrained to 1 mm in the z‐direction. Color codes for the layers of the ARSD are white = spunlace, blue = PETG, and purple = polyethylene

**FIGURE 2:**
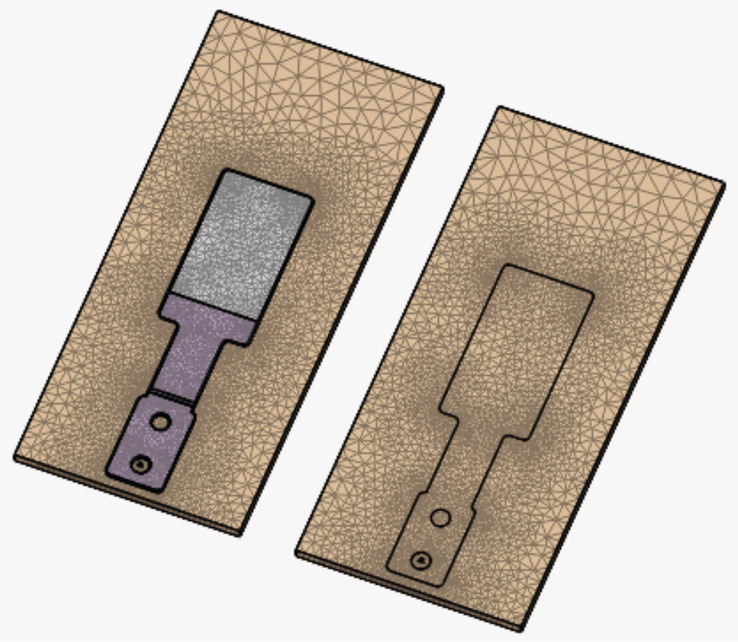
Schematic showing the mesh used for the finite element analysis and the mesh refinement employed around the stress concentration locations

A quasi‐static FEA model was created to compare the stress distribution and relative deformation around suture penetration sites in simulated skin samples both with and without the use of an ARSD. FEA was selected for this study because it is the method of choice for calculating the stresses involved in engineering designs and has had great impact in various biomedical applications,^12^, ^13^, ^14^ including the design of medical devices.^15^, ^16^, ^17^, ^18^ We refer the reader to various excellent sources for more information on FEA and its applications in medicine and medical devices.^15^, ^16^, ^17^, ^18^ A solid model of one case that included an ARSD and another that did not (Figure 1) was created using Onshape software (Onshape, Boston, Massachusetts), and the FEA was carried out using SimScale (SimScale GmbH, Munich, Germany). The finite element mesh was comprised of tetrahedral elements with refinement around the stress concentration locations (Figure 2) to give a total of 32,745 nodes. We refined the mesh until the stress values converged within ~0.6%. The FEA model included the point loading induced by the suture; however, the deformation of the suture was not modeled in the present study.

For both samples, a total tensile wound closure force of 25 N was applied for each case (Figure 1a,b) while the top edge of each skin sample was fixed (Figure 1c). The value of 25 N represents the upper end of typical wound closure forces based on in vivo human data.^19^, ^20^, ^21^, ^22^ Additionally, the skin displacement out of the plane was constrained to be 1 mm in the z‐direction to represent the realistic situation of the skin minimally pulling away from the site of a clinical wound closure (Figure 1d). The ARSD was modeled based on the same commercially available device that is used in our perfusion study (HEMIGARD ARS device, SUTUREGARD Medical Inc., Portland, Oregon). The ARSD consists of a layered polymer structure containing three materials: spunlace, polyethylene, and glycol‐modified polyethylene terephthalate (PETG). The design of the ARSD includes spunlace that runs the whole length of the device, a layer of PETG covering the spunlace at the end with the two holes, and a polyethylene top layer that covers the PETG and extends over some of the spunlace (see Figure 1 for a color‐coded depiction of the layers). All of the materials were considered to be homogeneous, isotropic, and linear elastic, and the material properties used for each layer and the skin are given in Table 1. Thickness values for the ARSD layers were taken to exactly match the device. All values for skin were estimated based on literature values.^23^, ^24^ While it is recognized the polymer layers and skin behave viscoelastically and viscoplastically under stress,^21^, ^25^, ^26^ using simple elastic properties is considered a suitable methodology to compare the worst‐case scenario that occurs upon initial loading before any stress relaxation can occur. Finally, the model assumed good adhesion between each material layer of the ARSD and also between the ARSD and the skin layer such than no separation between layers, or between the ARSD and the skin, occurred.

**TABLE 1:** Layer thicknesses and material properties used in the FEA model

Materials	Layer thickness	Elastic modulus	Poisson's ratio
Spunlace	0.24 mm	1 MPa	0.49
PETG	0.51 mm	2110 MPa	0.43
Polyethylene	0.08 mm	180 MPa	0.46
Simulated skin	1.90 mm	1.05 MPa	0.49

### 2.2 Perfusion study

We conducted a single‐center, randomized split‐scar comparison trial between August and October 2020. The split‐scar model was selected to reduce uncontrollable variables and has been used previously to compare suturing techniques.^27^ The Samaritan Regional Medical Centre Institutional Review Board in Corvallis, Oregon, approved this registered study (SHS IRB20‐054). Seventeen patients were enrolled in the study, and all patients gave written informed consent prior to enrollment. Inclusion criteria were male and female patients over 18 years of age presenting for surgical excision of cutaneous tumors on the trunk or extremities at an outpatient clinic in Corvallis, OR. Patients with linear closures longer than 3.0 cm were included. Study participant characteristics are summarized in Table 2, and wound dimensions are summarized in Table 3.

**TABLE 2:** Details on study participants and wound locations

Patient and wound attributes	Number of patients (percent of total)
Male	14 (82%)
Female	3 (18%)
Average age, in years (standard deviation)	75.9 (12.9) years
Body site	
Leg	4 (24%)
Arm	7 (41%)
Back	6 (35%)
Body side	
Left	4 (24%)
Right	8 (47%)
Midline	5 (29%)

**TABLE 3:** Details on average wound dimensions

Dimension measured	Average (standard deviation)
Original wound width	2.6 (0.6) cm
Original wound length	4.7 (0.8) cm
Final wound closure length	5.1 (0.9) cm

**FIGURE 3:**
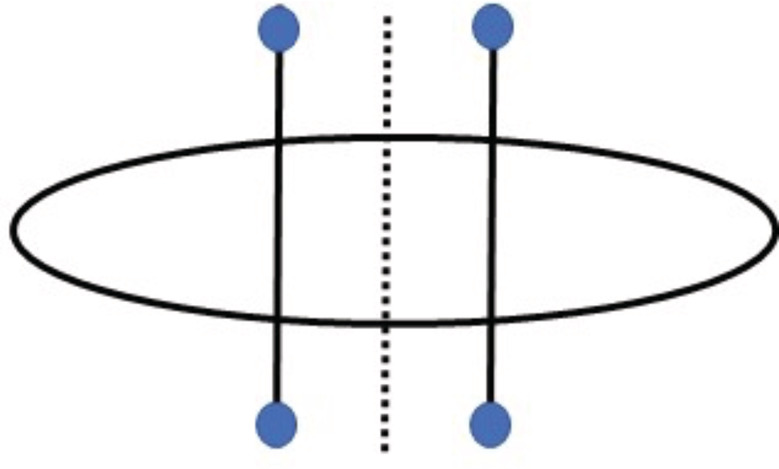
Percutaneous suture entry and exit points were marked 1 cm lateral to midline (dotted vertical line) and 1 cm from wound edges as indicated by blue circles. The two solid vertical lines represent the two 3‐0 nylon sutures used to approximate the wound

All procedures were performed by the same surgeon. Each surgical ellipse was marked with a long axis parallel to relaxed skin tension lines. Each fusiform specimen was removed with a number 15 scalpel blade, and minimal electrosurgery was performed. Wounds were divided in half, with the midline marked with a sterile surgical marking pen. Then, marks were made to indicate 1 cm from the midline on each side of the wound. On each of these lines, a marking was made 1 cm from the wound edge on each side of wound as depicted schematically in Figure 3.

The side to receive the ARSD was randomized using a random number generator. A transparent ARSD variant of the commercially available HEMIGARD was used to enable visualization of the entire wound. This ARSD differed from the commercial ARSD in that the spunlace layer (Figure 1) was made from polyurethane instead of nonwoven polyester. The ARSD was adhered to the skin on each side of the wound so that the holes were 1 cm from the wound edge. Size 3‐0 nylon sutures were used to close each side of the wound, one side with the ARSD and one side without.

A laser speckle contrast analysis (LASCA) system consisting of a camera (PeriCam NR; Perimed Inc; Las Vegas, Nevada) and software (PIMSoft, Perimed Inc; Las Vegas, Nevada) was used to capture recordings of perfusion units (PUs) of the skin surrounding the wound with both sutures tied. The camera was positioned orthogonal to the wound and adjusted to have a working distance of approximately 10 cm from the skin surface. Image capture was at 2.4 images per second with a resolution of 0.12 mm and high point density. Regions of interest (ROIs) were defined for each suture using a 1‐cm diameter circle. A time of interest (TOI) of at least 15 seconds was defined as a period of time with no patient movement in which the tracing of PUs was constant. Mean PUs within each ROI and TOI were recorded. After perfusion measurement, the wounds were closed in a standard linear fashion.

Statistical analysis was done by first confirming the normality of the data using the Shapiro‐Wilk test and then using a paired Student's *t*‐test to compare the mean values for the groups with and without the ARSD. Prism 8 software for Windows (GraphPad Software; San Diego, CA) was used for all statistical tests, and *p* ≤ 0.05 was considered statistically significant.

## 3 RESULTS

### 3.1 FEA model results

**FIGURE 4:**
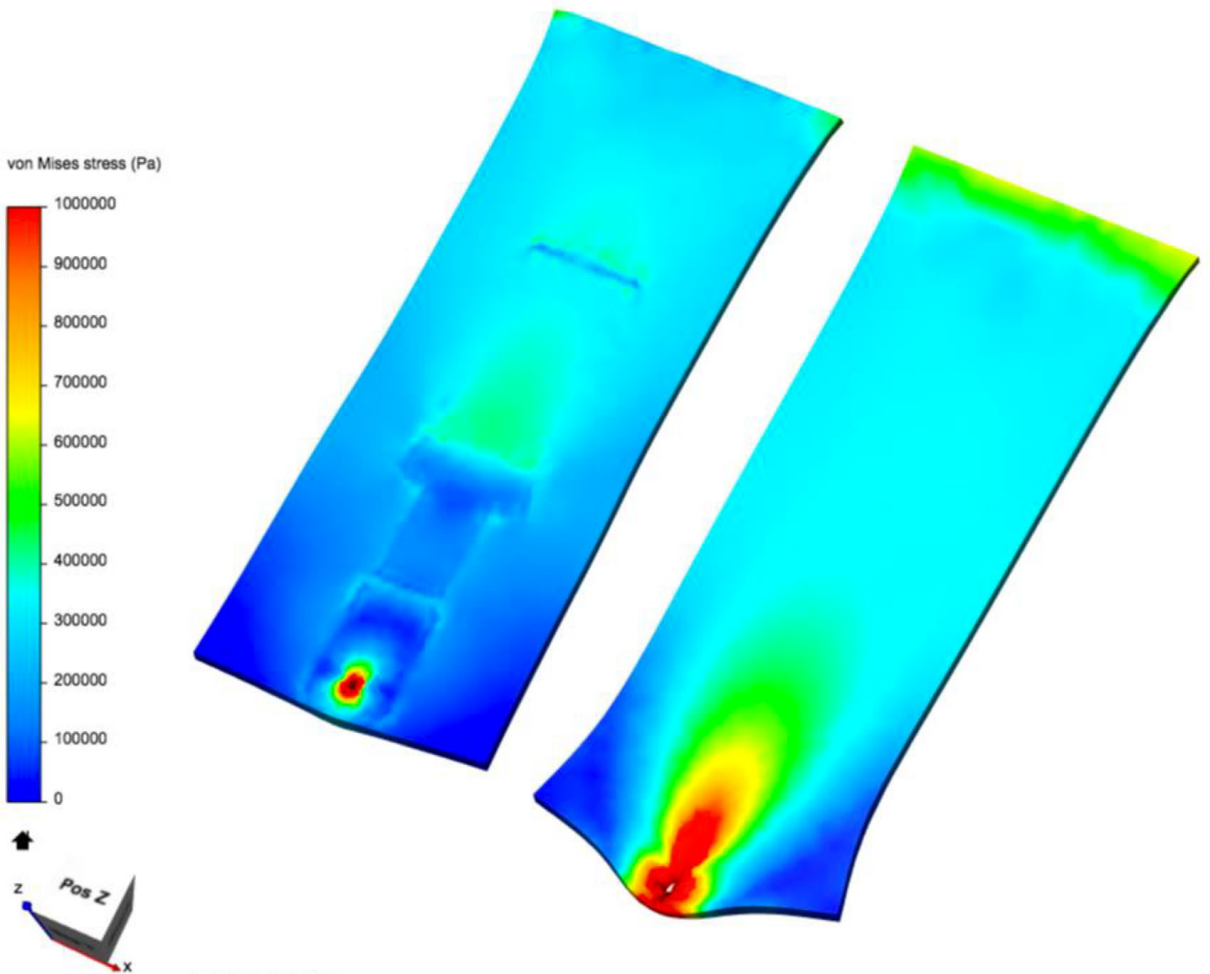
FEA results comparing skin samples with (at left) and without (at right) the adhesive retention suture device. The color scale depicts the von Mises stress level from zero (blue) to 1,000,000 Pascals (red)

Figure 4 shows the von Mises stress distribution for the two samples with and without the ARSD. From these images, it is clear that there is a high local stress region around the suture site for both samples. However, the bonding of the ARSD to the skin allows for the load transfer to the skin to occur over a larger area, thus reducing the local stress and deformation in the skin around the suture site. We performed our subsequent in vivo perfusion tests based on the hypothesis that by having less skin under high stress (ie, less red in Figure 4), we would find enhanced perfusion for the ARSD case.

### 3.2 Perfusion results

**FIGURE 5:**
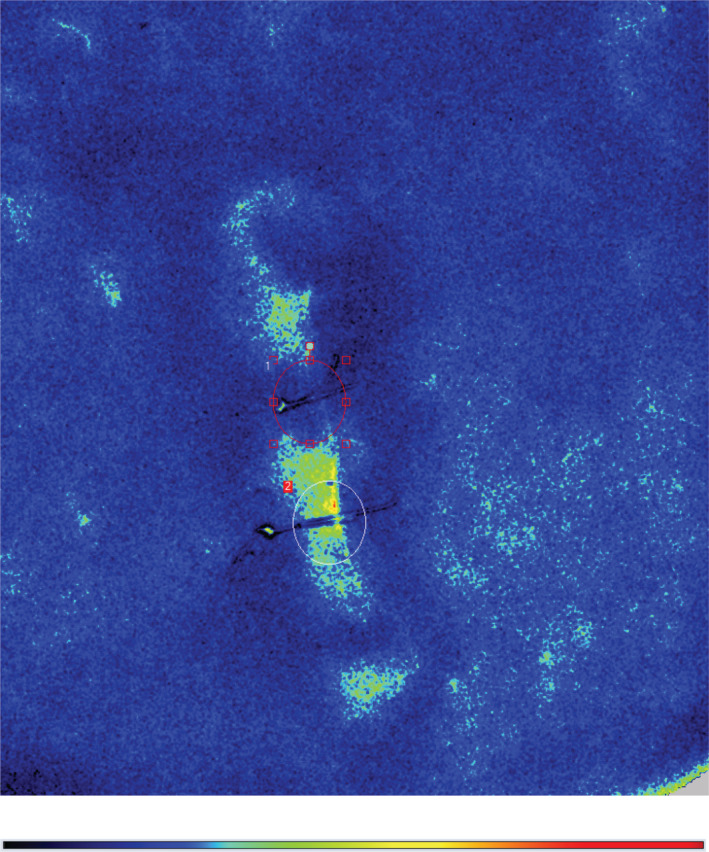
Laser speckle contrast analysis perfusion image captured during the closure of elliptical wound and color legend below. Dark blue indicates the lowest perfusion and red indicates the highest perfusion. Local tissue injury and local anesthesia result in increased blood supply at the wound edges. Region of interest 1 (red circle) is a suture alone showing reduction of perfusion to a mean of under 49 PU. Region of interest 2 (white circle) is a suture bolstered by the ARSD with a mean of 88 PU. The distance between the centers of the circles is 2 cm

A representative LASCA image is shown in Figure 5. In Figure 5, the red circle indicates the location of a suture alone, showing a reduced perfusion with an average measured value of under 49 PU. In contrast, the white circle shows a suture bolstered by the ARSD where the brighter colors indicate enhanced perfusion, and the average value was quantified to be 88 PU. Considering all of the wounds together, the perfusion at the wound edge in the ARSD‐protected wounds showed larger average perfusion of 52 ± 23 PU vs 42 ± 16 PU without the ARSD, and this mean improvement of 25% was statistically significant (*p* = 0.002).

## 4 DISCUSSION

The results of this study indicate a statistically significant 25% increase in perfusion at the time of wound closure with the use of an ARSD. Such results are consistent with prior studies that have shown how various suturing patterns can affect perfusion at the wound edge.^28^ Furthermore, the FEA results suggest that the lower perfusion for the bare suture is correlated with the higher concentration of local stress around the suture site. Thus, our hypothesis that having less skin under high stress (red in Figure 4) would give enhanced perfusion for the ARSD case was not rejected.

Wound dehiscence and delayed healing remain problems in cutaneous wound repair and are associated with increased costs and morbidity.^29^, ^30^ Inadequate closure of some lower extremity wounds, including pretibial lacerations in the elderly, is associated with increased mortality.^31^ Surgical procedures below the knee, such as saphenous vein graft harvesting and limb amputation, have an elevated risk of wound healing complications including dehiscence and edge necrosis.^30^, ^32^ In foot and ankle surgery, wound dehiscence is the most common postoperative complication, affecting approximately 5% of all cases.^33^ In orthopedic surgery, dehiscence occurrence over internal hardware results in further procedures, morbidity, and cost. Considering the importance of adequate perfusion in avoiding wound healing complications,^5^, ^6^, ^7^ it should be expected that the 25% improvement in perfusion found in this study could reduce postoperative complications such as edge necrosis and dehiscence, especially in zones of the closure where tension is highest. The ARSD also has the advantages of bolstering skin that is fragile to prevent “cheese wiring” of the skin and elevates the suture above the skin surface to prevent suture ingrowth.^11^, ^34^ Compared with other skin support methods such as using medical tape,^35^ Steri‐Strips,^36^ or polyethylene film,^37^ the ARSD has a designed‐for‐purpose multilayer, reinforced polymer construction that provides both stiffness and support without tearing or blistering the skin under applied stresses. The leading edge with holes where the sutures pass through is reinforced with both PETG and polyethylene (Figure 1) to withstand high suture tension without tearing. Meanwhile, the trailing edge of spunlace is stretchy to prevent shear force blistering on frail skin.

One limitation of this study is that the average age of patients in this study was slightly over 75 years. However, the data are highly relevant since the elderly are at higher risk of wound complications after lower limb procedures and worsened morbidity in the event of complications.^31^ A further limitation is that 85% of patients in this study were male. Thus, while the female patients in this study had 32% improved perfusion with the use of the ARSD, due to small number of female patients in this study, a subgroup statistical analysis was not performed.

Avoiding complications such as wound dehiscence and delayed healing remains a clinical challenge for the closure of some lower extremity wounds. Examples include pretibial lacerations in the elderly and wounds arising from orthopedic surgeries, saphenous vein graft harvesting, and limb amputation. The ability of an ARSD to reduce the local stresses in the skin and enhance perfusion around the suture site suggests the potential benefit in enabling more efficient healing while also supporting skin that is fragile to prevent skin tearing or “cheese wiring.”

## FUNDING

No financial support was received for this work.

## CONFLICT OF INTEREST

William Lear is co‐founder and chief technical officer for SUTUREGARD Medical, Inc., the manufacturer of the adhesive retention suture device used in this study. Jamie Kruzic and Jared Bahm act as scientific and engineering consultants for SUTUREGARD Medical, Inc. Allison Stoecker and Karsten Johnson have no conflicts of interest to declare.

## AUTHOR CONTRIBUTIONS

Conceptualization: Allison Stoecker, William Lear, Karsten Johnson.

Investigation: Allison Stoecker, William Lear, Jared Bahm.

Formal Analysis: William Lear, Jamie J. Kruzic.

Writing—Original Draft Preparation: William Lear.

Writing—Review and Editing: Allison Stoecker, William Lear, Karsten Johnson, Jared Bahm, Jamie J. Kruzic.

All authors have read and approved the final version of the manuscript.

Jamie Kruzic had full access to all of the data in this study and takes complete responsibility for the integrity of the data and the accuracy of the data analysis.

## TRANSPARENCY STATEMENT

Jamie Kruzic affirms that this manuscript is an honest, accurate, and transparent account of the study being reported; that no important aspects of the study have been omitted; and that any discrepancies from the study as planned (and, if relevant, registered) have been explained.

## Data Availability

The data that support the findings of this study are available from the corresponding author upon reasonable request.
